# Decoding the “Pain-to-Door” interval using psychological, medical, and demographic factors: A multi-center study

**DOI:** 10.1371/journal.pone.0325140

**Published:** 2025-06-30

**Authors:** Hamidreza Roohafza, Feridoun Noohi, Sara Bagherieh, Marjan Mansourian, Media Babahajiani, Hamidreza Marateb, Mohammadjavad Alemzadeh, Ansari, Aboozar Fakhr Mousavi, Mohammad Mehdi Peighambari, Masoumeh Sadeghi

**Affiliations:** 1 Isfahan Cardiovascular Research Center, Cardiovascular Research Institute, Isfahan University of Medical Sciences, Isfahan, Iran; 2 Cardiovascular Intervention Research Center, Rajaie Cardiovascular Medical and Research Center, Iran University of Medical Sciences, Tehran, Iran; 3 School of Medicine, Isfahan University of Medical Sciences, Isfahan, Iran; 4 Department of Automatic Control, Biomedical Engineering Research Center, Universitat Politecnica de Catalunya, BarcelonaTech (UPC), Barcelona, Spain; 5 Department of Biostatistics and Epidemiology, Isfahan University of Medical Sciences, Isfahan, Iran; 6 Biomedical Engineering Department, Engineering Faculty, University of Isfahan, Isfahan, Iran; 7 Cardiovascular Diseases Research Center, Department of Cardiology, Heshmat Hospital, School of Medicine, Guilan University of Medical Sciences, Rasht, Iran; 8 Heart Valve Disease Research Center, Rajaie Cardiovascular Medical and Research Center, Iran University of Medical Sciences, Tehran, Iran; 9 Cardiac Rehabilitation Research Center, Cardiovascular Research Institute, Isfahan University of Medical Sciences, Isfahan, Iran; UN Mehta Institute of Cardiology and Research Center, INDIA

## Abstract

**Background:**

Reducing the amount of time between the onset of symptoms and presentation to a healthcare facility, namely the “pain-to-door” interval, is of utmost importance in patients with myocardial infarction. In the present study, we aimed to shed light on the psychological, medical, and demographic factors that are associated with this vital time, and the details of this association.

**Methods:**

We used the baseline data of 1685 participants from a 3-year, multi-centric, cohort study. The pain to door time was estimated as the interval between symptoms’ onset and arrival at the hospital. Patients were asked to fill out valid and reliable questionnaires regarding sociodemographic factors, depression, health anxiety, type D personality, sense of coherence, coping strategies, and quality of life. Data was then analyzed to attain the p-value and hazard ratios (HR) of different variables.

**Results:**

In the multivariate analysis, being male (HR: 0.81, 95% CI: 0.68–0.98) and a history of angina (0.82, 0.69–0.96) were associated with shorter pain-to-door durations. A history of diabetes mellitus also made the cut marginally (p-value: 0.059). On the contrary, health anxiety (1.27, 1.09–1.49), history of depression (1.57, 1.21–2.05), high socioeconomic status (1.25, 1.03–1.51) and sense of coherence (1.34, 1.14–1.57) scores were associated with longer pain-to-door durations.

**Conclusion:**

Our findings demonstrate that personal, social, and economical characteristics play a pivotal role in determining patients’ pain-to-door time duration. Screening high-risk individuals in terms of the factors that tend to increase the pain-to-door time alongside educating people and healthcare providers on the importance of this interval and its contributing factors must be a priority considering the devastating burden of CVDs.

## Introduction

Ischemic heart diseases (IHDs) affected over 125 million people in 2017, and the number is projected to grow drastically [1]. Furthermore, this global leading cause of death is also responsible for a significant load of morbidity and disability, posing huge personal, social, and economical burden to the society [2]. Numerous efforts have been undertaken to quell the morbid consequences of IHDs including refining the triage systems and preventing extensive cardiac damage by educating high-risk individuals to seek professional help within the golden time [2–4]. However, these attempts have often failed to come to fruition and mitigate the burden of CVDs.

Consequently, emphasizing the importance of a crucial interval, namely “Pain-to-door”, should not be neglected, as the amount of time that it takes myocardial infarction (MI) patients to seek professional help following the onset of chest pain plays a pivotal role in determining the prognosis [3–5]. The shorter the pain-to-door time is, the higher are the chances of preserving left ventricular vitality and decreasing the subsequent mortality [3]. Long symptom onset to first medical contact, another term used interchangeably with pain-to-door time, consumes the majority of total ischemic time [4]. On the contrary, the “Door-to-Balloon” time has been effectively reduced in most regions of the world due to advancements of medical apparatus and hospital facilities [5].

Various factors contribute to the amount of pain-to-door time, including personal factors such as level of education, occupation, presence of psychological problems, e.g., depression, anxiety, etc., and social factors such as place of residence, mean annual income, insurance coverage, etc. Among all the contributor of prolonged pain-to-door time, personal factors have frequently been overlooked, with only few studies delving into the complexity of the relationship between demographic factors and psychological problems [4,6].

To the best of our knowledge, the only available study on the association of psychological variables and pain-to-door time, attempted to compare the contributing factors between a high and low-middle income country, and reported that higher educational level and the presence of bystanders in the scene were significantly associated with lower pain-to-door time among STEMI patients [7]. Nonetheless, the study lacks generalizability as it does not elaborate on the presumable effects of other potentially-confounding factors. Moreover, the diverse demographic factors of STEMI patients are repeatedly disregarded in the proposed guidelines on STEMI management.

Due to the high prevalence of IHDs [1], the scarcity of data on the contributing correlates of prolonged pain-to-door time, and the importance of shortening this time to help patients receive timely care, we have designed this multi-center cohort study to investigate the demographic, psychological, and medical aspects of STEMI patients to which prolonged pain-to-door time can be attributed. To the best of our knowledge, this is the first study to include psychological contributes of the pain-to-door interval, which can in turn lead to the identification of barriers in delivering the best and most on-time care to patients and provide practical solutions to this global surging crisis.

## Methods

### Study design

The present study uses the baseline data retrieved from a 3-year, multi-centric, cohort study that aims to achieve adequate data to design the first cardiovascular risk assessment model according to behavioral, psychosocial and traditional factors in patients with ST-segment elevation myocardial infarction (CRAS-MI). Details on the methodology, inclusion and exclusion criteria, and statistical analysis of the mentioned cohort project have been previously explained [8].

The CRAS-MI study was conducted between 2018–2020 in 5 major Iranian provinces including Tehran, Isfahan, Yazd, Hormozgan, and Gilan [8]. The inclusion criteria of CRAS-MI were as follows: age of 18–75, a diagnosis of STEMI according to the American College of Cardiology/American Heart Association guidelines for the first time, and a signed informed consent [8,9]. The exclusion criteria were: history of cardiovascular diseases, history of a severe illness with an estimated lifetime fewer than 3 years, refusal to signed the informed consent, ongoing participation in another research study, and inability to self-care [8]. The data of the CRAS-MI study was obtained using patients’ medical records and standardized questionnaires.

Nevertheless, participation in the present study required a complete set of baseline medical records, as well. Consequently, not all 1707 CRAS-MI subjects were able to enroll in this project, leading to a final participants count of 1685. The required data to performed this study was retrieved on June 20, 2023.

The present study has been approved by the Ethical Committee of Isfahan University of Medical Sciences (Ethics code: IR.MUI.MED.REC.1402.056). All participants signed written, informed consent forms, as well. The authors did not have access to information that could identify individual participants during or after data collection.

### Tools & measurements

#### Pain to door time.

Once a patient is brought to one of the recruited hospitals in this study [8], a trained nurse asks them about the exact time the symptoms have begun, regardless of whether or not the patient chooses to participate in the study afterwards. In case the patient is unconscious or not lucid enough to remember the exact time of symptoms’ onset, an accompanying family member and/or friend is asked regarding the matter. The time of arrival at the hospital is also written on the patients’ medical records. The pain to door time is estimated as the interval between symptoms’ onset and arrival at the hospital. This interval is then categorized into two subgroups: lower and higher than 12 hours, as it has been mentioned in previous studies that PCI is usually performed within the first 12 hours after the onset of pain in clinical settings [10].

#### Socio-demographic factors.

Obtained demographic variables are sex, age, marital status; married or unmarried; and educational level, by asking the years of education. Years of education will be reported within the following categories: 0–5 years, 6–12 years, and ≥ 12 years of education. Furthermore, socioeconomic status is assessed by socioeconomic status questionnaire (SES). With scores ranging from 0 to 17 the results of this 6-item questionnaire will be later classified into three subgroups: low, middle, and high. The items include a) head of households’ education level, b) head of households’ employment status, c) number of rooms in the house, d) car ownership, e) using notebook, laptop, or tablet in the house, and f) whether or not the family has fun, pleasure, or travel abroad [11].

#### Medical history.

Questions regarding past medical history of diabetes mellitus, hypertension, angina, depression, and family history of CVDs are asked. Moreover, patients BMI levels are calculated using their heights and weights, which are measured in the hospital once the patients are stable enough to stand up straight without help. BMI is then categorized into the following classes: underweight and normal (<25), overweight (25–29.9), and obese (≥ 30).

#### Depressive symptoms.

Consisting of essential nine criteria on which DSM-IV depressive disorder diagnosis is based, patient health questionnaire-9 (PHQ-9) is a self-administered instrument to diagnose depressive disorders. The scores of each of the nine criteria range from 0 (not at all) to 3 (nearly every day), with the total score ranging from 0 to 27 [12]. Cronbach’s α coefficient of the Farsi version is 0.88, and one-week test-retest reliability is 0.79 [13,14].

#### Type D personality.

The type D scale-14 (DS-14) is a useful instrument used to assess negative affectivity, social inhibition, and type D personality [15]. Within the Farsi version of DS-14, test-retest reliability of the negative affectivity, and the social inhibition subscales are 0.86 and 0.77, respectively, with the Cronbach’s α coefficients of these scales being 0.84 and 0.86, respectively [16,17].

#### Sense of coherence.

The short form of sense of coherence questionnaire is filled out by the patients. This questionnaire comprises of 3 components on a 7-category semantic differential scale: a) comprehensibility (5 items), b) manageability (4 items), and c) meaningfulness (4 items), with a score ranging from 13 to 91 [18]. The Iranian version of this questionnaire has a remarkable Cronbach’s alpha of 0.77 [19].

#### Coping strategies.

A comprehensive, multicomponent, self-administered coping strategies questionnaire was utilized to evaluate the coping potential of the participants when faced with stressful life events. The questionnaire had 23 items, which were categorized into five subscales: a) positive reinterpretation and growth, b) problem engagement, c) acceptance, d) seeking support, and e) avoidance. The reliability of the questionnaire was determined using Cronbach’s alpha coefficient (a = 0.84). Each of the 23 items were scored on a 3-point scale (never = 0, sometimes = 1, and often = 2), leading to separate, overall scores being reported for each subscale [20]. Moreover, the Iranian version of this questionnaire manifested satisfactory validity and reliability [21].

#### Quality of life (QoL).

The 12-item short form survey (SF-12) for quality of life, a shorter version of SF-36, calculates a physical component score and a mental component score for the estimation of quality of life using the weighted means of the eight domains. The scores are later subclassed into three categories: low (score range: 17–23), medium (score range: 24–36), and high (score range: 37–44) [22].

#### Distress score.

Kessler psychological distress scale (K6), a 6-item questionnaire about a person’s emotional state, is filled out by the participants to assess their distress score. The scores of this questionnaire range from 0 to 24 [23]. Higher scores indicate higher distress levels.

#### Health anxiety.

A domain of the diagnosis criteria of psychosomatic research (DCPR), healthy anxiety questionnaire consists of 4 yes/no questions and is used in the present study to estimate the level of health anxiety among the participants [24]. Ultimately, the results will be presented as a Yes or a No to the question of “Does the patients have health anxiety?”.

### Statistical analysis

#### Descriptive statistics.

Categorical variables in the study are presented as frequencies and percentages. For the analysis of these variables, the chi-squared test is employed. Continuous variables, where applicable, are expressed as medians along with their interquartile ranges (IQR). The Shapiro-Wilk test is used to evaluate the normality of the data distribution. The Mann-Whitney U test is applied for continuous variables that do not follow a normal distribution.

#### Regression analysis.

Univariable and multivariable Cox regression models are utilized to identify factors predicting the “pain-to-door” time (categorized by a 12-hour threshold). The dependent variable in these models is the time to arrive at the hospital after the initial 12-hour period. The independent variables (predictors) include age, gender, marital status, level of education, socioeconomic status, body mass index (BMI), medical history (angina, depression, hypertension, diabetes), family history of cardiovascular diseases (CVDs), sense of coherence, quality of life, type D personality, depressive symptoms, distress score, and health anxiety. Brier Score, as a measure to evaluate the performance of the model, the C index criteria based on the data were used to compare the performance of Cox models for checking the predictive ability to distinguished between early and late presentation of the event. It should be noted that for computing the evaluation criteria, all variables were included in both models. Given the testing of multiple psychosocial variables, we controlled for the false discovery rate using the Benjamini-Hochberg (B-H) procedure. Both unadjusted and FDR-adjusted p-values are reported to ensure the robustness of the findings.

#### Statistical software and significance.

The analyses are performed using R statistical software (version 4.1.1). All statistical tests are two-sided, and a p-value of less than 0.05 indicates statistical significance.

## Results

### Participant demographics

This study analyzed 1685 patients, all with complete information, an average age of 56.20 years, ranging from 50.00 to 63.00 years. The sample exhibited a male predominance, with only 313 (18.6%) female participants. Table 1 provides a comprehensive overview of the participants’ basic characteristics.

**Table 1:** Background factors and clinical characteristics of the study population stratified by Pain to door ≤ 12 h and > 12 h.

	All N = 1685	Pain to door time≤12 h N = 1242	Pain to door time>12 h N = 443	P _value
**Sex** (Female)	313(18.6%)	217(17.47%)	96(21.7%)	0.051
Age	56.20(50.00−63.00)	56.21(50.00−63.00)	56.15(49.00−63.00)	0.878
**Marital status** (Married)	1414(87.9%)	1054(84.86%)	360(81.26%)	0.330
**Education**				<0.001
0−5 y	551(33.9%)	377(30.35%)	174(39.27%)	
6−12 y	779(47.9%)	591(47.58%)	188(42.43%)	
≥ 12 y	297(18.3%)	238(19.16%)	59(13.31%)	
**Socioeconomic status**				0.120
Low	380(23.3%)	268(21.57%)	112(25.28%)	
Middle	776(47.5%)	575(46.29%)	201(45.37%)	
High	477(29.2%)	366(29.46%)	111(25.05%)	
**Body mass index**				0.714
<25	637(37.8%)	468(37.68%)	169(38.1%)	
25−29.9	769(45.6%)	573(46.1%)	196(44.2%)	
≥30	279(16.6%)	201(16.18%)	78(17.6%)	
**History of angina**	276(16.5%)	203(16.34%)	73(16.4%)	0.960
**History of Depression**	103(6.1%)	75(6.03%)	28(6.3%)	0.830
**History of hypertension**	597(35.5%)	440(35.42%)	157(35.4%)	0.965
**History of diabetes**	424(25.2%)	303(24.39%)	121(27.3%)	0.216
**Family history of CVD**	748(44.6%)	547(44.01%)	201(45.37%)	0.537
**Sense of coherence**				0.727
Low/ Medium	475(29.1%)	349(28.1%)	126(28.44%)	
High	1156(70.9%)	859(69.16%)	297(67.04%)	
**Quality of Life**				0.175
Low	45(2.8%)	28(2.25%)	17(3.80%)	
Moderate	1260(77.3%)	934(75.20%)	326(73.58%)	
High	326(20.0%)	245(19.72%)	81(18.2%)	
Type D personality	806(49.4%)	591(47.58%)	215(48.53%)	0.527
**Depression**	775(47.5%)	570(45.89%)	205(46.27%)	0.680
Distress score	7.66(4.00−11.00)	7.54(4.00−11.00)	7.98(3.00−12.00)	0.239
**Health anxiety**	307)18.9% (	236(19.00%)	71(16.02%)	0.198

### Pain-to-door time analysis

**Fig 1:**
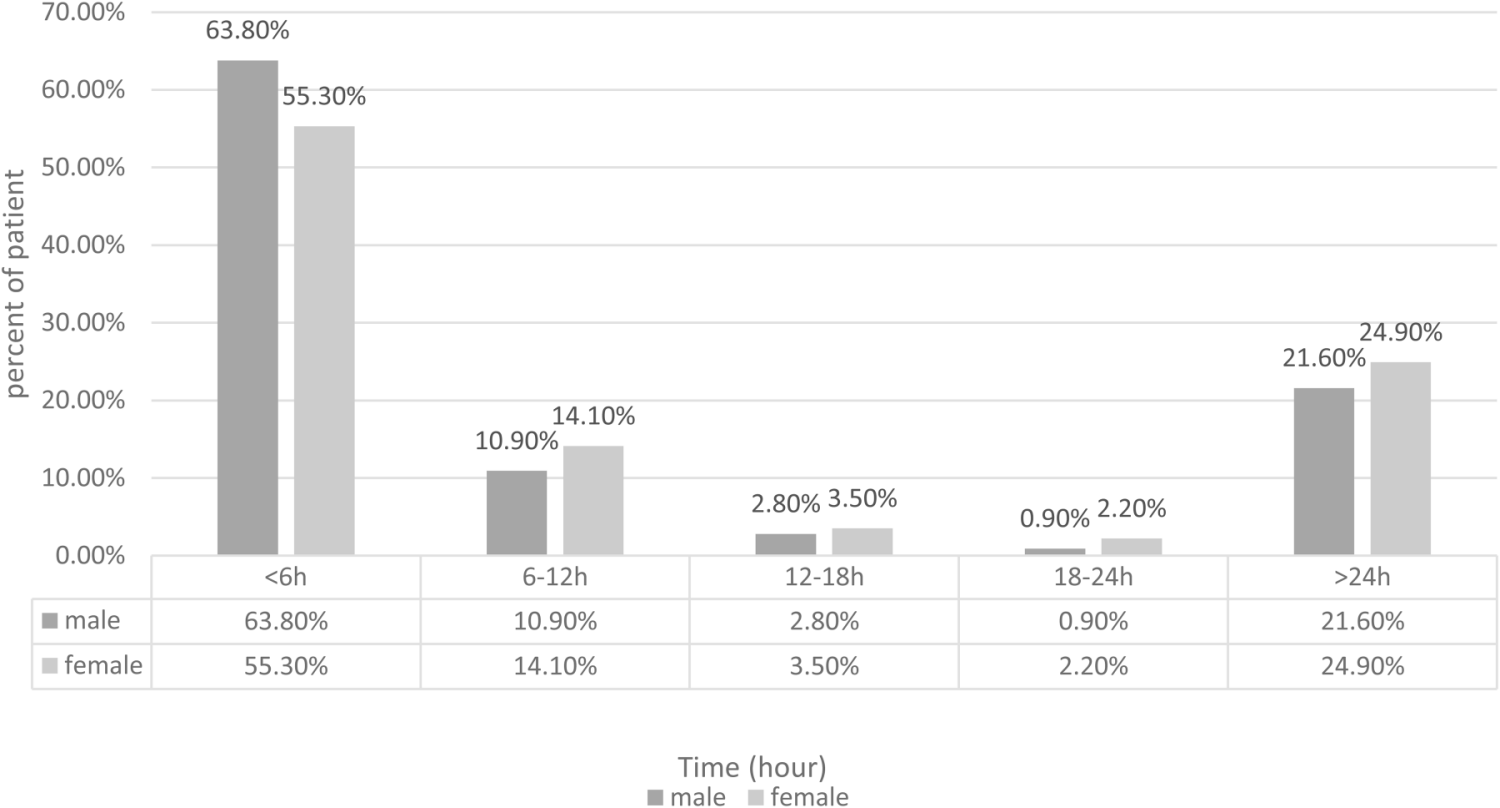
Percentage of female vs. male patients that presented the emergency department within the first day of symptoms’ onset.

A notable finding was the variation in educational levels between patients with different pain-to-door times. Patients with a pain-to-door time under 12 hours generally possessed higher educational levels, a statistically significant difference (p < 0.001). Fig 1 displays the distribution of male versus female patients concerning their emergency department arrival time post-symptom onset. Interestingly, a higher percentage of male patients (1025, 74.7%) presented to the emergency department within 12 hours compared to female patients (217, 69.4%), though this difference narrowly missed statistical significance (p = 0.051, Table 1).

In order to enhance the clinical interpretability of our findings, we calculated the median pain-to-door times in hours for several key subgroups. For instance, female patients had a median delay of 5.0 hours compared to 3.68 hours for male patients, reflecting an absolute difference of 1.33 hours. Likewise, educated patients showed a median pain-to-door time of 3.0 hours, contrasting with a delay of 4.01 hours among non-educated patients (an absolute difference of 1.01 hours). Additional comparisons revealed that married patients arrived about 0.27 hours earlier than unmarried patients, patients with angina experienced a 0.63-hour longer delay, and those with diabetes had a 0.37-hour delay relative to non-diabetic patients. Other factors, including health anxiety and socioeconomic status, were also associated with differences in the range of 0.1–1.06 hours. Overall, these findings provide a more tangible perspective on the effect sizes, thereby reinforcing the clinical implications of our study.

### Univariate and multivariate analysis

**Fig 2:**
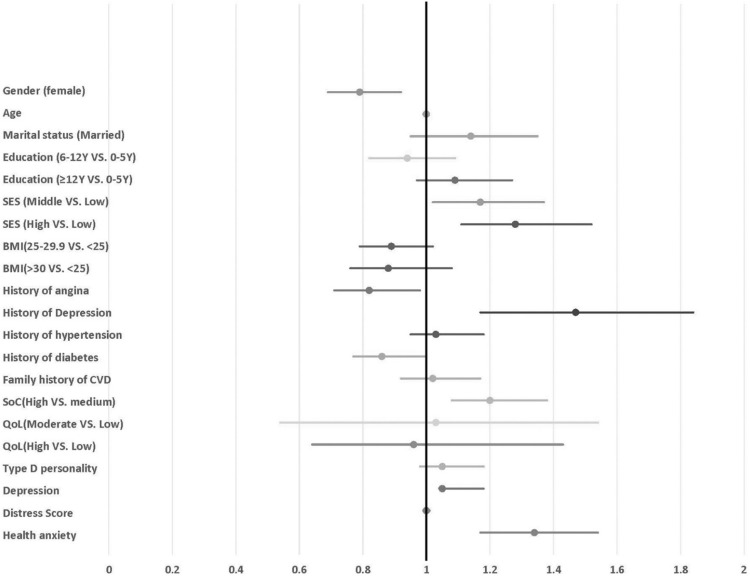
Univariate Cox regression analysis of risk factors on pain to door time. **SES**: Socioeconomic Status, **BMI**: Body Mass Index, **SOC**: Sense of Coherence, **QoL**: Quality of Life.

Considering the baseline similarities of the groups, both univariate and multivariate hazard ratio (HR) analyses were conducted for the group with pain-to-door times below 12 hours. The results are detailed in Fig 2 and Table 2. In the multivariate analysis (Table 2), being male (HR: 0.81, 95% CI: 0.68–0.98) and having a history of angina (HR: 0.82, 95% CI: 0.69–0.96) were significantly associated with shorter pain-to-door times. Additionally, a history of diabetes mellitus showed a marginally significant association (p = 0.059). Conversely, factors such as a history of depression (HR: 1.57, 95% CI: 1.21–2.05), health anxiety (HR: 1.27, 95% CI: 1.09–1.49), high socioeconomic status (SES) (HR: 1.25, 95% CI: 1.03–1.51), and high sense of coherence (SOC) scores (HR: 1.34, 95% CI: 1.14–1.57) correlated with longer pain-to-door durations. Notably, current depression was also linked to increased pain-to-door time in the univariate analysis (HR: 1.47, 95% CI: 1.17–1.88). The C-index for our model is 0.72, indicating a good predictive ability to distinguish between early and late presentations of the event. To address the potential for Type I error due to multiple comparisons, we applied the Benjamini-Hochberg FDR correction. While several predictors maintained statistical significance after adjustment—such as History of Depression (FDR-adjusted p = 0.0042), Sense of Coherence (High) (FDR-adjusted p = 0.00231), and Health Anxiety (FDR-adjusted p = 0.014)—other predictors that were close to the significance threshold in the unadjusted analysis did not meet the adjusted significance level. The updated results, presented in Table 2, include both unadjusted and FDR-adjusted p-values to provide a clearer assessment of the associations.

**Table 2:** Multivariate adjusted Cox regression analysis of risk factors on pain to door time.

	Hazard Ratio	95% CI	Unadjusted P _value	FDR-adjusted p-values
**Sex (male)**	0.81	(0.68−0.98)	0.033	0.115
Age	0.99	(0.99 −1.00)	0.909	0.954
**Marital status** (Married)	1.00	(0.81−1.23)	0.966	0.966
**Education**				
0-5 y	Ref			
6-12 y	0.92	(0.80−1.05)	0.237	0.413
≥ 12 y	1.10	(0.93−1.30)	0.256	0.413
**Socioeconomic status**				
Low	Ref			
Middle	1.12	(0.94−1.32)	0.176	0.410
High	1.25	(1.03−1.51)	0.020	0.084
**Body mass index**				
<25	Ref			
25-29.9	0.92	(0.81−1.05)	0.255	0.413
≥30	0.89	(0.74−1.06)	0.203	0.413
**History of angina**	0.82	(0.69− 0.96)	0.017	0.084
**History of Depression**	1.57	(1.21−2.05)	<0.001	<0.001
**History of hypertension**	1.07	(0.94 −1.22)	0.282	0.423
**History of diabetes**	0.86	(0.74−1.00)	0.059	0.177
**Family history of CVD**	1.00	(0.89−1.14)	0.886	0.954
**sense of coherence**				
Low/medium	Ref			
High	1.34	(1.14−1.57)	<0.001	<0.001
**Quality of life**				
Low	Ref			
Moderate	0.80	(0.52−1.23)	0.322	0.450
High	0.68	(0.43−1.08)	0.109	0.286
**type D personality**	1.06	(0.91−1.22)	0.421	0.552
**Depression**	1.05	(0.90−1.22)	0.508	0.627
**Distress score**	0.99	(0.98−1.01)	0.813	0.948
**Health anxiety**	1.27	(1.09−1.49)	0.002	0.014

## Discussion

The present study evaluates the association between psychological, medical, and demographic factors and pain-to-door time. In general, the result indicates that the existence of psychological comorbidities such as health anxiety and depression are significantly associated with longer pain-to-door intervals. Furthermore, it demonstrates that certain medical and demographic factors, e.g., history of angina and diabetes mellitus, can contribute to the amount of time it takes patients to seek professional care following the onset of symptoms, as well. Such factors have not been widely investigated before, and grasping a deeper understanding of them may hold the potential of impressively reducing post-MI morbidity and mortality.

In the context of MI, time is a pivotal prognostic tool. Despite the advancements in interventional cardiology that have revolutionized MI care and treatment, the time window between the onset of vessel blockage and the beginning of irreversible damage is limited. A timely management of an MI patient results in a faster reperfusion of the ischemic myocardium, leading to a dramatic reduction in further complications [25,26]. As such, the importance of reducing the intervals between symptoms’ onset to admission and admission to therapy cannot be overstated [27]. In spite of medical breakthroughs that have effectively reduced the interval of admission to therapy, pain-to-door time is still highly neglected as a potential factor leading to delayed access to care in MI patients. This mentioned time should not be viewed as a single contributing factor, but rather a combination of psychological, medical, and demographic factors that impact the pain-to-door time in a delicate manner and via complex interplays.

According to a recent meta-analysis, a cluster of DM patients have the “irritable nociceptor” profile [28], in which hyperalgesia, reduced pressure pain threshold, and overactive pain perception is present [28–30]. Consequently, DM might elevate patients’ sensitivity to pain, particularly to pressure pain. Similarly, it is proposed that a history of angina inclines patients to favorable outcomes in future episodes of angina. This can be attributable to patients’ familiarity with the quality of the pain, a state of angina hypervigilance, and being more likely to have caretakers on a regular basis [31,32].

Being male was another factor that shortened the pain-to-door interval in this study. The extensive body of literature in this area suggests the male sex functions as protective factor against adverse cardiac events due to less symptoms’ complexity, lower prevalence of atypical pain, and better access to healthcare services can facilitate the reduction in pain-to-door interval [33]. Additionally, the occurrence of non-cardiac chest pain is much more frequent in women than men [34], gradually increasing their threshold to identifying a chest pain episode as cardiac.

On the contrary to the aforementioned factors that encourage the patients to present to the emergency department faster, there are other variables that are associated with longer pain-to-door intervals and can deteriorate patients’ outcomes following an episode of MI. For instance, our results show that depression and health anxiety can lengthen this interval, which is in accordance with the findings of previous studies [35]. These studies have demonstrated that depression and pain can form a vicious cycle, where pain aggravates the symptoms of depression, and depression can give rise to feelings of pain all over the body [36]. As a result, patients with depression are commonly told that their disorder is associated with spurious or exaggerated feelings of pain that may not always have an organic root, which can make the patients indifference to an actual episode of cardiac chest pain [37]. Likewise, in individuals with anxiety, chest pain, dyspnea, diaphoresis, and other symptoms of cardiac ischemia accompanies 20% to 70% of panic attacks [36]. Repeated episodes of anxiety and such attacks, which mimic cardiac chest pain symptoms, can make the patients apathetic towards episodes of chest pain that originate from the heart, as seen with depression [38]. Moreover, anxiety provokes functional impairment that hinders a person’s ability to approach an emergency swiftly and effectively, hence the longer pain-to-door intervals [8].

Another interesting finding of the present study was the inverse association of SES score and pain-to-door time, meaning that socioeconomically-disadvantaged people are more likely to have a shorter pain-to-door interval. Although more investigations are required to illuminate the details underpinning this situation, we suggest that individuals with lower SES scores are more likely to have comorbidities that can make them susceptible to myocardial infarction [39]. Quite similarly, lower scores in the SOC scale were associated with a shorter pain-to-door interval. SOC allows individuals to be more resilient, stay stable, and maintain their quality of in spite of daily stressors. One of the key components of SOC is manageability, the extent to which people deduce they have control over stressors on their own and possess sufficient resources to satisfy their needs and tackle their problems [40].

The present study is the first to evaluate the relation between psychological factors and pain-to-door interval. A previously-published study [6] reports relatively similar findings regarding demographic, psychological, and medical contributing factors; however, fails to appreciate the importance of psychological variables. Furthermore, our study enrolls participant from multiple centers across the nation, which not only increases the samples size, but also reduces the possibility of suffering biases by using structured questionnaires. Notwithstanding these strengths, the major limitation of this project is that it still highly depends on the answers provided by the patients in response to the checklists and questionnaires. Furthermore, patients’ severity of symptoms, distance to hospitals, and whether or not they had used any medication prior to EMS arrival were not recorded and analyzed. Consequently, further research is called-for to shed light on the intricate details of this escalating problem.

## Conclusion

In conclusion, the present study demonstrates how psychological, medical, and demographic factors affect pain-to-door time in patients with STEMI. Namely, being male, a history of angina, and a history of DM were associated with shorter pain-to-door interval, whereas a history of depression, health anxiety, higher SES and SOC scores were observed more frequently in patients with a longer pain-to-door interval. Since a relatively short pain-to-door time is vital for ensuring the highest post-MI recovery and reducing subsequent mortality and morbidity, the findings can help identify high-risk patients in terms of long pain-to-door time, and educate them regarding the symptoms of acute MI, in an attempt to help minimize the amount of time between symptoms’ onset and presentation to the emergency department.
